# The influence of corn trypsin inhibitor on the contribution of coagulation determinants to the Technoclone Thrombin Generation Assay (TGA) and the Calibrated Automated Thrombogram (CAT)

**DOI:** 10.1371/journal.pone.0263960

**Published:** 2022-02-25

**Authors:** Carolina E. Touw, Ype de Jong, Astrid van Hylckama Vlieg

**Affiliations:** 1 Department of Clinical Epidemiology, Leiden University Medical Center, Leiden, the Netherlands; 2 Department of Orthopaedic Surgery, Leiden University Medical Center, Leiden, the Netherlands; 3 Department of Internal Medicine, Leiden University Medical Center, Leiden, the Netherlands; Karolinska Institutet, SWEDEN

## Abstract

In thrombin generation (TG) assays, regarded as global coagulation tests, contact activation is considered a major problem which can be eliminated by adding Corn Trypsin Inhibitor (CTI). In previous studies, however, venous thrombosis risk prediction using TG assays did not improve after CTI addition. However, it is unknown whether CTI addition could help to detect subtle but relevant nuances in determinants of TG, making the assay more suitable to detect disturbances in the coagulation system. This study’s objective was to assess whether the addition of CTI is associated with a broader contribution of individual coagulation factors to the total amount of thrombin formed in Calibrated Automated Thrombogram (CAT) and Technoclone Thrombin Generation Assay (TGA). Thrombin generation was measured in 326 healthy individuals from THE VTE study at very low tissue factor concentrations, with and without addition of CTI prior to blood sampling. The influence of several coagulation factors on total amount of thrombin formed, i.e. area under the curve (AUC) or endogenous thrombin potential (ETP), was analysed using multiple linear regression with standardisation of all values resulting in Z-scores with 95% confidence intervals (95%CI). Association between coagulation factors and TG changed minimally after addition of CTI. Largest changes after CTI addition were found for following factors: for CAT: free protein S (from 0.00 (95%CI -0.12 to 0.12) to -0.29 (95%CI -0.43 to -0.15)) and protein S (from -0.05 (95%CI -0.18 to 0.08) to -0.21 (95%CI -0.37 to -0.05)); for TGA: antithrombin (from -0.11 (-0.23 to 0.02) to -0.19 (-0.30 to -0.07)), factor VIII (from 0.15 (0.03 to 0.27) to 0.24 (0.13 to 0.36)) and fibrinogen (from 0.12 (-0.01 to 0.26) to 0.19 (0.06 to 0.32)). In conclusion, there is no clear trend towards a broader contribution of coagulation factors in samples handled with CTI compared with those handled without CTI.

## Introduction

Prothrombin time (PT) and activated partial thromboplastin time (APTT) are two tests which are used routinely in clinical practice to assess potential coagulation disorders. These tests are adequate to assess deficiencies of procoagulant factors, but they do not give a sufficient impression of the overall coagulation in vivo, i.e. including the compensation of anticoagulant factors [1]. Most of the tests appeared sensitive only to very specific abnormalities as these tests are based on clot-time, which only gives information regarding the time of clot initiation. Thrombin generation tests also include the features of the thrombin generating process after the clotting has occurred, which means that the balance between both procoagulant and anticoagulant factors is assessed. Therefore a thrombin generation test is regarded as a global coagulation assay and allows for a more sensitive and comprehensive detection of coagulation deficiencies in vivo [2–4].

Modest changes of procoagulant factors and the contribution of natural anticoagulant mechanisms are undetectable when anything other than very low tissue factor (TF) concentrations are used to initiate thrombin generation. However, when very low TF concentrations are used, measurements of hypercoagulability become imprecise. It was shown that this is due to contact factor activation during sample collection and processing [5]. This artefact can be abolished by sampling blood directly into tubes with anticoagulant containing corn trypsin inhibitor (CTI), an inhibitor of activated factor XII. Using CTI it is possible to measure the kinetics of thrombin generation and resultant fibrin polymerisation rates in response to very low TF concentrations with a high degree of precision [5]. Therefore, despite the fact that the thrombin generation test has a high potential for use in a clinical setting, there are some aspects which need to be optimized before it can be used as a tool to diagnose coagulation disorders [6].

Previous studies failed to find a clear effect of CTI on the predictive value of thrombin generation assays on venous thrombosis risk [5, 7–10]. Currently, it is unclear how CTI affects the contribution of individual determinants of thrombin generation. Potentially, subtle nuances in coagulation factor levels, which are relevant to thrombin generation and the risk of venous thrombosis, are masked by contact activation. Removing contact activation by addition of CTI might expose these nuances, thereby creating a more global representation of the coagulation system.

The aim of our study is to assess whether the addition of CTI to a thrombin generation assay is associated with a broader contribution of individual coagulation factors to the total amount of thrombin formed, thereby making it a more global assay. There are two commonly used assays which measure thrombin generation: Calibrated Automated Thrombogram (CAT) and Technoclone Thrombin Generation Assay (TGA). In this study, the effect of CTI was tested in both the CAT and TGA assay.

## Materials and methods

### Subjects

The study population consisted of 531 healthy individuals (223 men, 308 women) who were the control subjects of THE VTE (**T**hrombophilia, **H**ypercoagulability and **E**nvironmental risks in **V**enous **T**hrombo**E**mbolism) study [10]. Inclusion took place between March 2003 and December 2008. Patients with DVT of the leg or a PE were identified in both Leiden and Cambridge. Patients’ partners, aged 18 to 75 years old, were invited to participate as control subject in both centres.

In current study, we only considered healthy individuals for inclusion, since we were interested in determinants of thrombin generation and not in the risk of venous thrombosis. Furthermore, subjects with Factor V Leiden (FVL) or prothrombin (PT20210A) mutation, those who did not provide blood samples, and those who used anticoagulants at the time of blood draw were excluded. We obtained approval of the local ethics committees in Leiden and Cambridge.

### Blood collection

All included subjects provided blood samples with and without addition of CTI, which were measured with both CAT and TGA assays. For the blood sample without CTI, i.e., the standard sample, venous blood was collected with a 21-gauge needle using minimal suction with a light tourniquet from the antecubital vein into Sarstedt Monovette tubes containing 0.106 mol/L trisodium citrate. For the blood sample with CTI, i.e., the optimized sample, additional samples were drawn directly into 3mL Sarstedt tubes containing 0.35mL of citrate/CTI; a mix of 0.3mL 0.106mol/L trisodium citrate (Sarstedt, Leicester, UK) and 0.05mL CTI (1.1mg/mL) resulting in a final CTI concentration of 18.3 μg/mL in whole blood. Of both standard and optimized blood samples platelet poor plasma was obtained by centrifugation one time for 10 minutes at 3000g at room temperature and stored at -70°C until it was analysed.

### Laboratory measurements

Thrombin generation was measured with Thrombinoscope™ CAT assay (Thrombinoscope BV, Maastricht, the Netherlands) and Technoclone TGA® assay (Technoclone Ltd, Vienna, Austria). Thrombinoscope® assay was performed, as previously described [11]. For the standard sample, 5pM TF trigger concentration (Dade Behring, Milton Keynes, UK) was used. For the optimized sample, the TF-trigger concentration was lowered to a concentration of 1pM instead of 5pM. At TF-concentrations <15pM, the factor XIIa-driven thrombin generation equals or even exceeds that of TF-driven thrombin generation [2].

Thrombinoscope™ software was used to convert the fluorescence signal into nMol/L thrombin activity with correction for the inner filter effect and thrombin bound to alpha-2 macroglobulin activity, as described by Hemker et al. [12]. TGA assay was carried out on the Ceveron® alpha coagulation analyser (Technoclone, Vienna, Austria) using Technothrombin® TGA reagents (Technoclone, Vienna, Austria). In TGA for the standard sample, 40μL of plasma was added to 10μL of an activation reagent (containing 17.9pM TF and 3.2 μM phospholipids) and 50mL of calcium-fluorogenic substrate reagent (15mM CaCl_2_, 1mM Z-G-G-R-AMC), resulting in a TF-concentration of 0.5pM. In the optimized sample with a CTI-concentration of 18.3 μg/mL, the TF-concentration was the same. There was no software correction for inner filter effect or alpha-2 macroglobulin activity. Thrombin generation, expressed as nM/min, was reported as endogenous thrombin potential (ETP) and area under the curve (AUC) for CAT and TGA, respectively.

Coagulation determinants protein C and S activity, free protein S, and activity of antithrombin III, factors II, VIII, IX, and XI, and fibrinogen were measured as activity levels with a mechanical clot detection method on a STA-R coagulation analyser (Roche Diagnostics, Almere, The Netherlands). All measurements were performed according to the instructions of the manufacturer (Diagnostica Stago, Asnières, France). Levels of FIX antigen were determined by ELISA. Screening for the FVL and PT20210A mutations was carried out on genomic DNA as previously described [13, 14]. For each assay, participants with missing values were excluded.

### Statistical analysis

**Fig 1:**
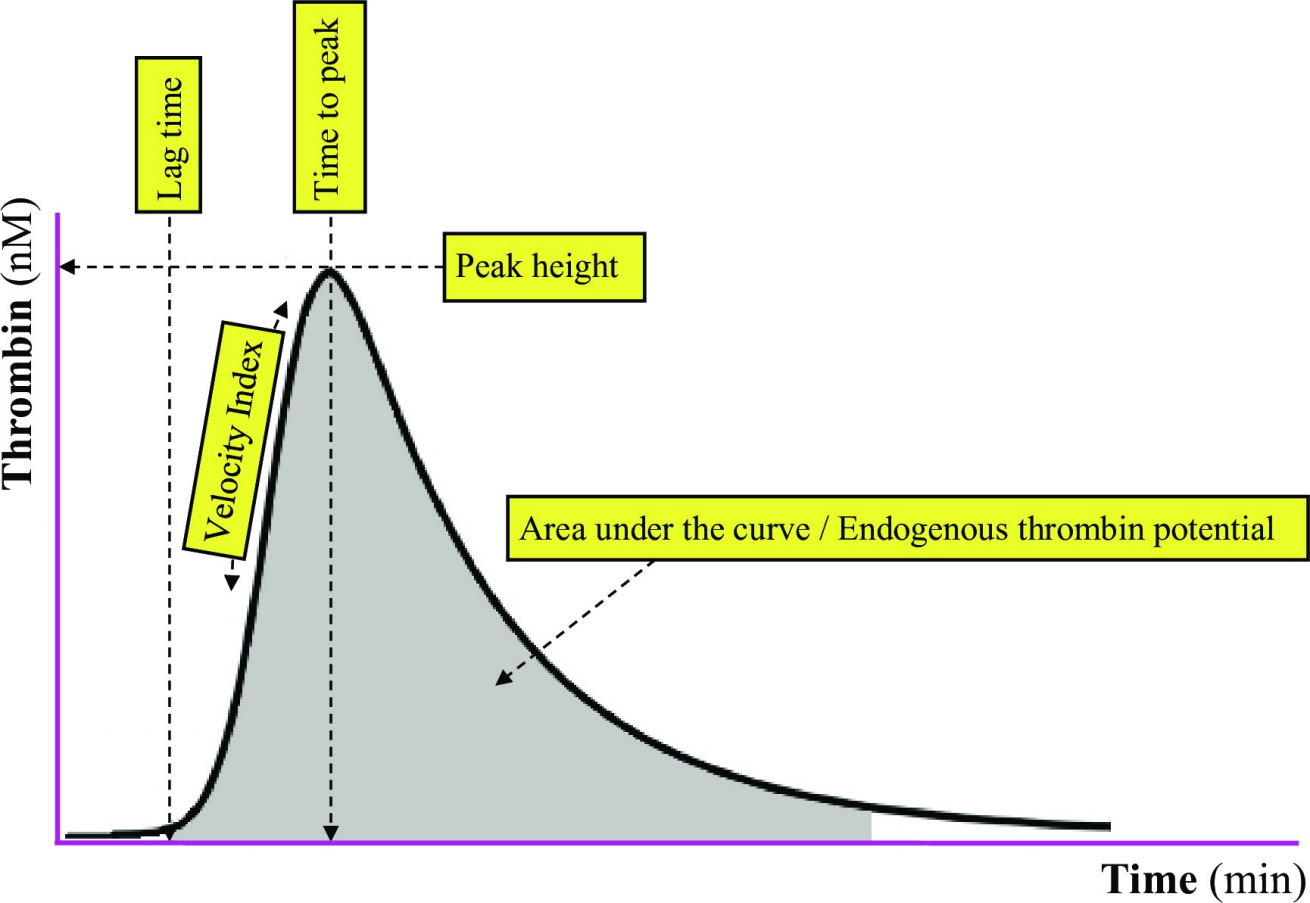
Overview of thrombin generation parameters.

All parameters of the CAT and TGA, both without and with addition of CTI, were described and expressed as means with 95% confidence intervals (95% CI). In order to assess the individual effect of coagulation factor levels on thrombin generation in the standard and optimized samples, we used multiple linear regression analysis. Additionally, determinants of other parameters of CAT and TGA, i.e. lag time, time to peak (ttP), curve steepness (V1) and peak height (for the TGA) were assessed in both samples. (Fig 1) Independent variables were protein C and S activity, free protein S, and activity of antithrombin III, factors II, VIII, IX, and XI, and fibrinogen, as well as demographic factors, i.e., sex (1 = men, 2 = women), age, oral contraceptive use (0 = no, 1 = yes) and smoking behaviour (0 = never or ever smoker, 1 = current smoker). Because of the difference in ranges of coagulation determinants and parameters of CAT and TGA, comparison was facilitated by standardizing these values (Z-scores). Multiple linear regression analysis was performed with the standardized values of thrombin generation parameters as well as standardized values of the independent variables. The resulting standardized regression coefficient β for a coagulation factor indicated the increase in standard deviation (SD) when that particular coagulation factor increased with 1 SD, while the other variables remained unchanged. The Z-scores were provided with 95% confidence intervals (95% CIs). SPSS 25.0 (IBM Corp. Released 2017. IBM SPSS Statistics for Windows, Armonk, NY) was used for all analyses.

## Results

### Demography

Table 1 shows the general characteristics of the study population. Of the 531 participants, 163 did not provide a blood sample and were therefore excluded from current analyses. Furthermore, 7 subjects used anticoagulants at the time of the blood draw and were excluded from analyses. Individuals with FVL (n = 16) or PT20210A mutation (n = 14), or those in whom these mutations were undetermined (n = 5) were excluded for all analyses. This resulted in inclusion of 326 healthy individuals (121 men and 205 women) in current analyses. The mean age of this study population was (54.6; 95% CI: 53.3 to 55.9), 60 (18.4%) of the participants were current smokers and 28 women (13.7% of all women) were using oral contraceptives.

**Table 1:** Demography of included control subjects of THE VTE study.

	All controls (n = 326)
**Age, mean (95% CI)**	54.6 (53.3 to 55.9)
**Sex, n (%)**	
Men	121 (37.1)
Women	205 (62.9)
**Smoking–current, n (%)**	60 (18.4)
**Contraceptive use–current, n (% of women)**	28 (13.7)

### Thrombin generation parameters

**Fig 2:**
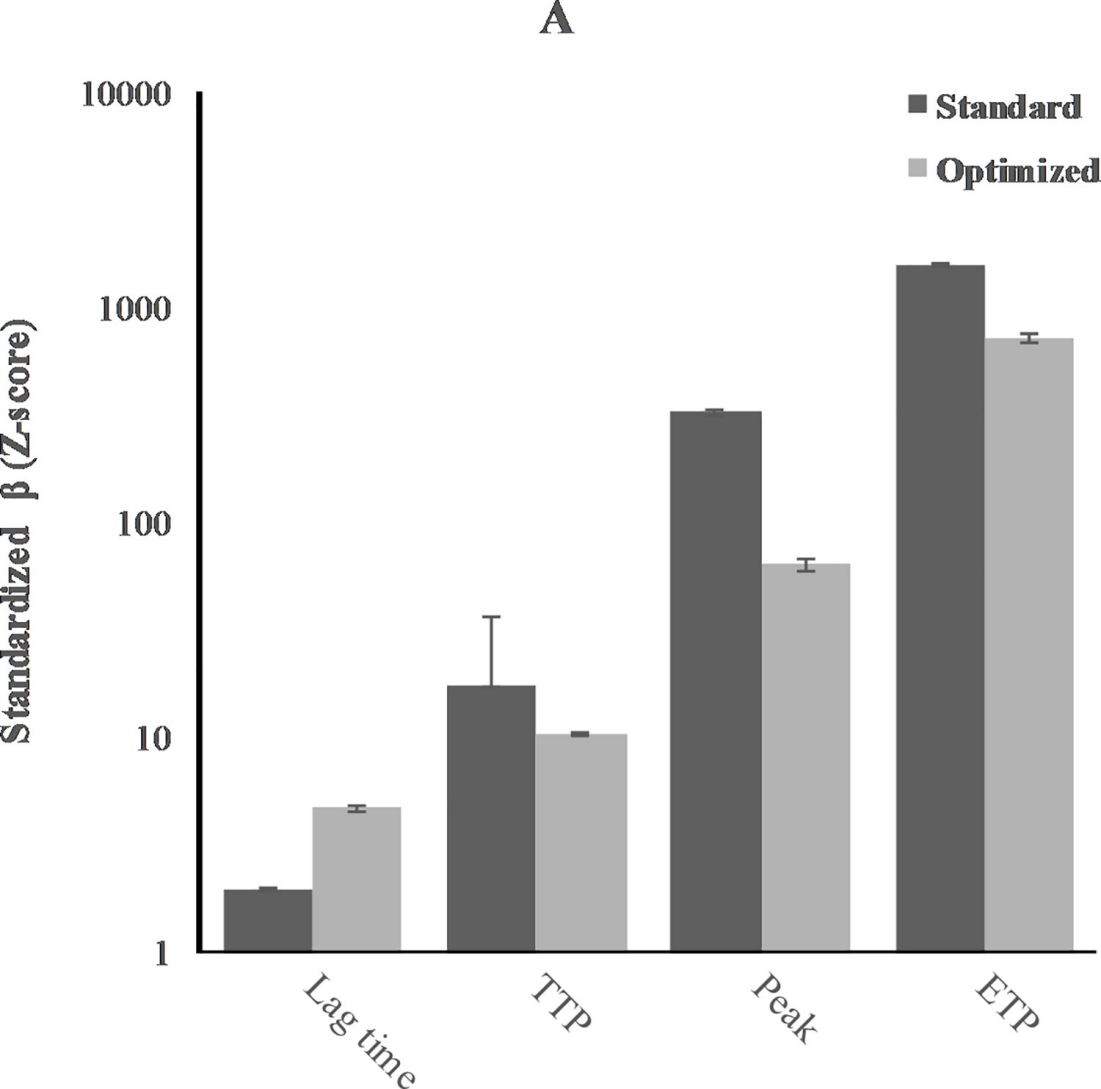
Effect of CTI on assay parameters for CAT.

**Fig 3:**
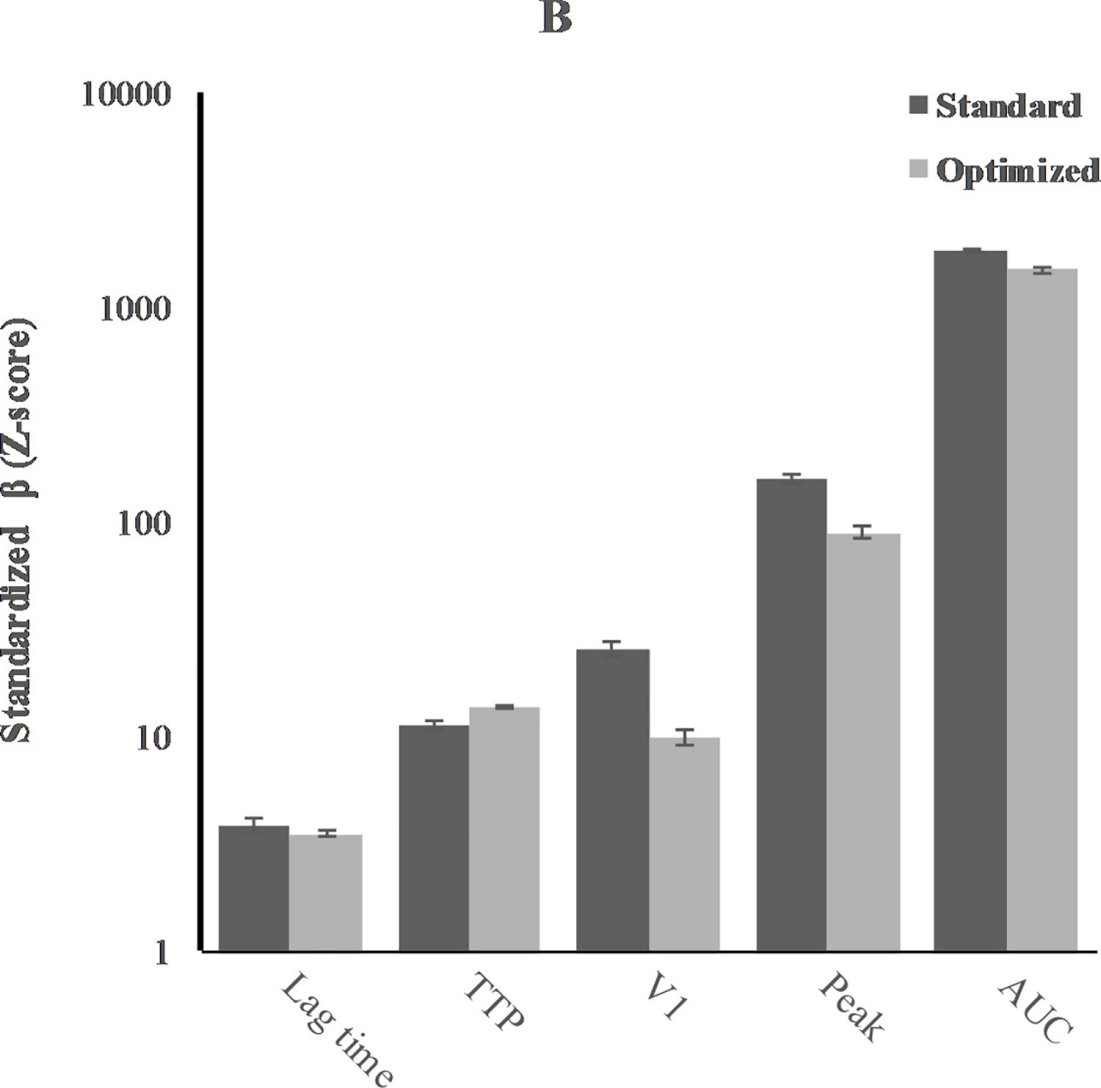
Effect of CTI on assay parameters for TGA.

Mean values of the assay parameters of the TGA (lag time, ttP, V1, peak, and AUC) and of the CAT (lag time, ttP, peak, and ETP) measured in standard and optimized samples are shown in Table 2, Figs 2 and 3. In standard samples, the thrombin potential measured with TGA was 1818nM·min (95%CI 1770 to 1866), while it was 1586nM·min measured with CAT (95%CI 1551 to 1621). Lag time measured with TGA was longer than measured with CAT: 3.81min (3.49 to 4.14) and 1.95min (1.91 to 2.00), respectively. Time to peak was 11.3min (10.7 to 11.9) in TGA and 17.4min (-1.98 to 36.7) in CAT. The peak height in TGA was 157nM (149 to 165), which was lower than in CAT (324nM, 317 to 331).

**Table 2:** Mean results with 95%CI in subjects for both Calibrated Automated Thrombogram (CAT) and Technoclone Thrombin Generation Assay (TGA).

Assay	Standard	Optimized (CTI)
	*Mean (95%CI)*	*Mean (95%CI)*
**CAT**		
Lag time *(min)*	1.95 (1.91 to 2.00)	4.71 (4.55 to 4.87)
Time to peak *(min)*	17.4 (-1.98 to 36.7)	10.4 (10.2 to 10.7)
Peak height *(nM)*	324 (317 to 331)	63.3 (58.9 to 67.7)
ETP *(nM·min)*	1586 (1551 to 1621)	716 (679 to 754)
**TGA**		
Lag time *(min)*	3.81 (3.49 to 4.14)	3.50 (3.37 to 3.64)
Time to peak *(min)*	11.3 (10.7 to 11.9)	13.6 (13.3 to 14.0)
V1 *(nM/min)*	25.8 (23.7 to 27.9)	10.0 (9.15 to 10.8)
Peak height *(nM)*	156 (148 to 164)	89.1 (84.2 to 94.0)
AUC *(nM·min)*	1818 (1770 to 1866)	1493 (1446 to 1540)

*Abbreviations*: ETP, Endogenous thrombin generation; V1, steepness of curve; AUC, area under the curve; CAT, Calibrated Automated Thrombogram (CAT); TGA, Technoclone Thrombin Generation Assay (TGA); CTI, Corn Trypsin Inhibitor.

As expected, addition of CTI clearly affected the parameters of both assays. In CAT, lag time increased from 1.95min (1.91 to 2.00) to 4.71min (4.55 to 4.87). When analysing the optimized samples, the peak height decreased from 324nM (317 to 331) to 63.39nM (58.9 to 67.7), with a corresponding decrease of thrombin generation, i.e. from 1586nM·min (1551 to 1621) to 716nM·min (679 to 754). In TGA, lag time remained the same, i.e. from 3.81min (3.49 to 4.14) in standard samples and 3.50min (3.37 to 3.64) in optimized samples. Time to peak increased subtle from 11.3min (10.7 to 11.9) to 13.6min (13.3 to 14.0). V1 decreased markedly from 25.8nM/min (23.7 to 27.9) to 10.0 (9.15 to 10.8) with a corresponding decrease of peak height: from 157nM (149 to 165) to 89.1nM (84.2 to 94.0). The AUC was also lower, 1818nM·min (1770 to 1867) in standard samples and 1493nM·min (1447 to 1540) in optimized samples.

### Contribution of coagulation factors to thrombin generation

**Fig 4:**
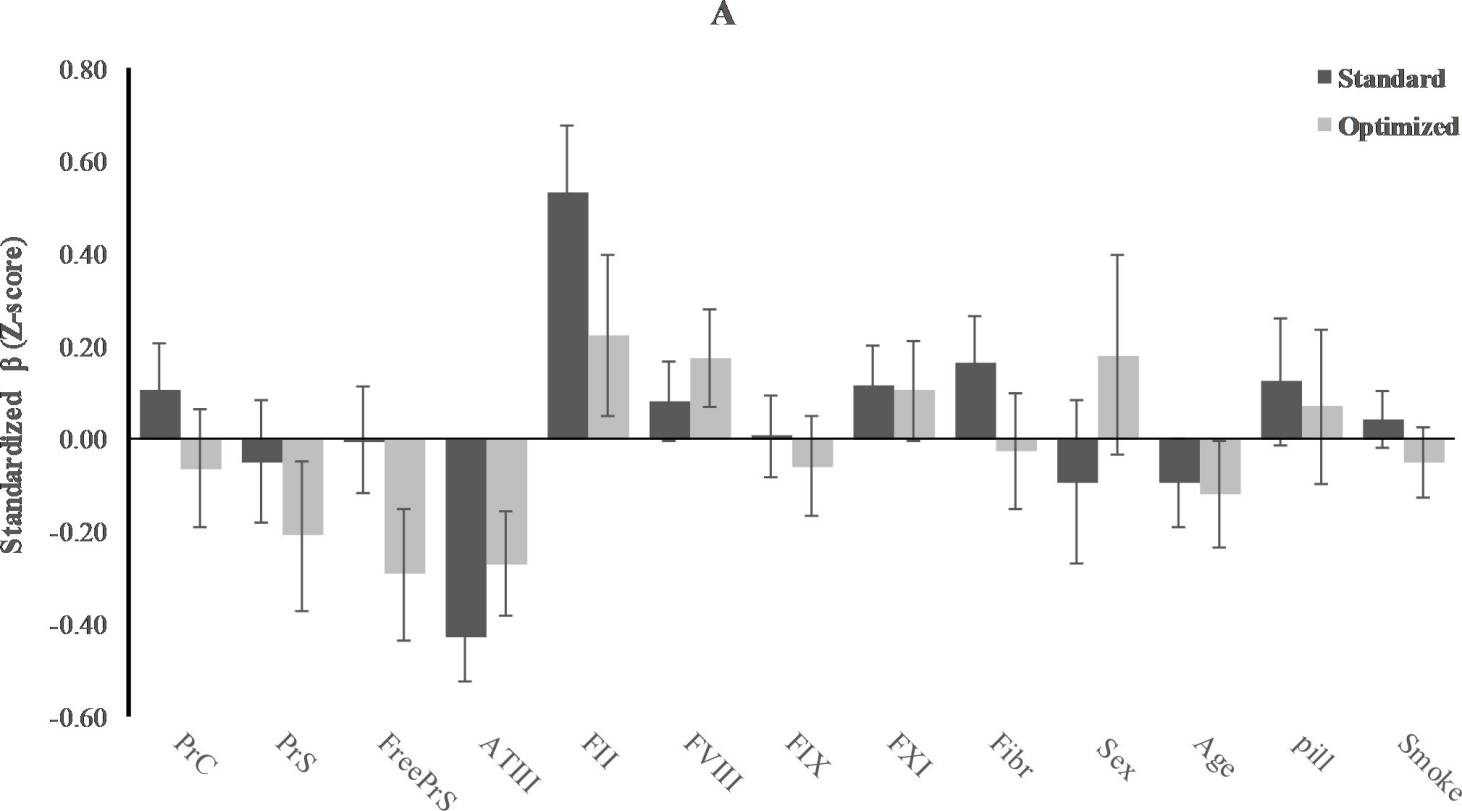
Effect of CTI on relative contribution of coagulation factors to thrombin generation measured with CAT. Mean change in thrombin generation with 1 standard deviation (SD) increase in coagulation determinants, while other determinants remain the same. Results for standard versus optimized sample.

**Fig 5:**
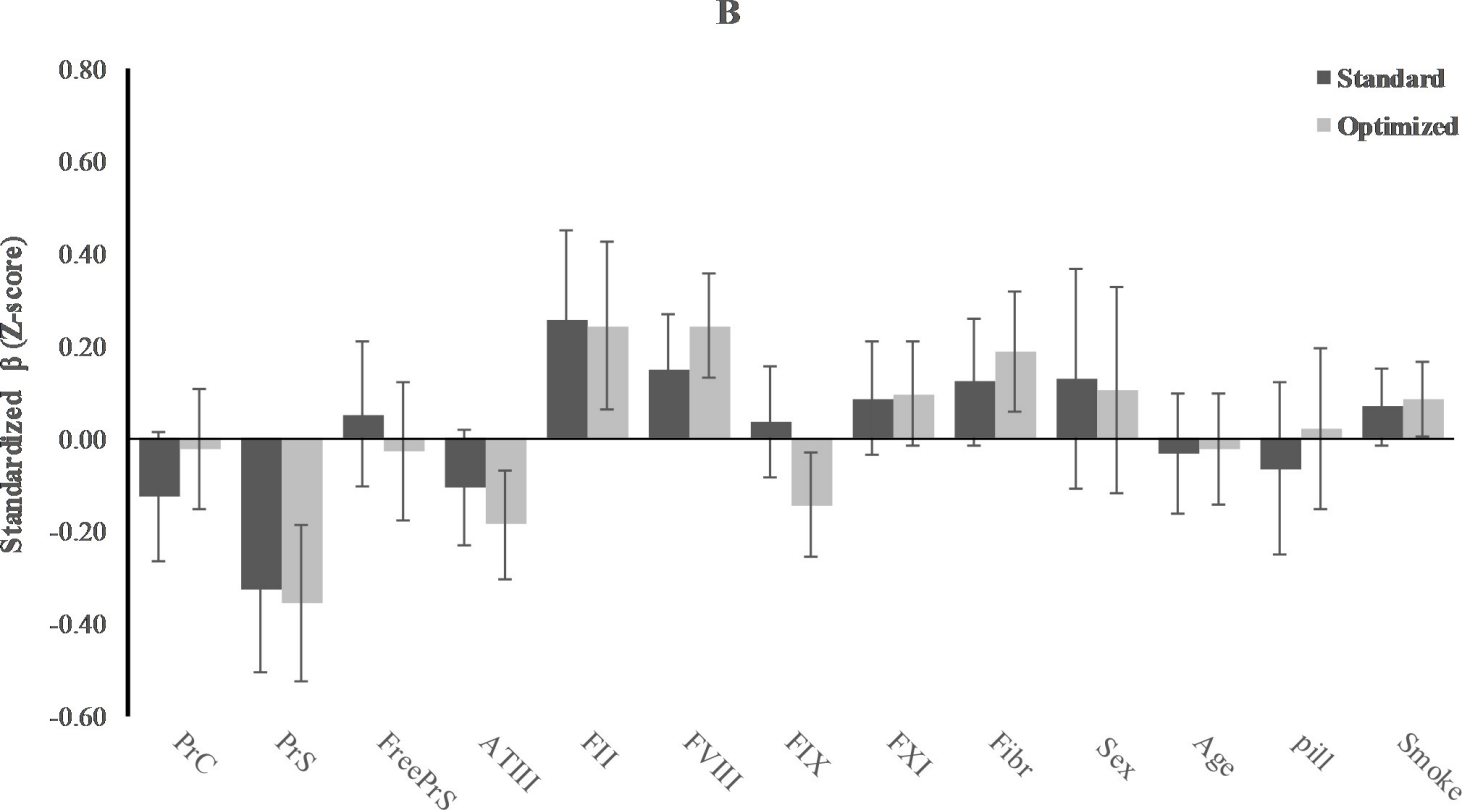
Effect of CTI on relative contribution of coagulation factors to thrombin generation measured with TGA. Mean change in thrombin generation with 1 standard deviation (SD) increase in coagulation determinants, while other determinants remain the same. Results for standard versus optimized sample.

In Table 3, Figs 4 and 5, the results of multivariate analysis of standardized values (Z-scores) of ETP (CAT) and AUC (TGA) measured in standard samples, taking into account the influences of sex, age, smoking, and oral contraceptive, are shown. Results of the determinants of the other parameters of the CAT and the TGA assays (i.e. lag time, time to peak, V1, and peak) are presented in the S1 and S2 Tables. In the standard samples, there were some minor differences in the determinants of TG measured with CAT or TGA. Whilst in both assays there seemed to be a broad influence of coagulation proteins on TG, without CTI there was no influence of free PS and IX. Strongest determinants of TG in the CAT assay were AT (Z-score: -0.43; 95%CI: -0.52 to -0.34) and FII (Z-score: 0.53; 0.39 to 0.68), while for the TGA these were PS activity (Z-score: -0.33; -0.51 to -0.15) and FII (Z-score: 0.25; 0.06 to 0.45).

**Table 3:** Mean change in thrombin generation with 1 standard deviation (SD) increase in coagulation determinants.

Standardized values (Z-score)	SD	CAT ETP (SD = 318.78)	CAT ETP + CTI (SD = 341.58)	TGA AUC (SD = 438.58)	TGA AUC + CTI (SD = 425.01)
		Standardized B (95% CI)			
Protein C activity	23.6	0.10 (0.00 to 0.21)	-0.06 (-0.19 to 0.06)	-0.13 (-0.27 to 0.01)	-0.02 (-0.16 to 0.11)
Protein S activity	15.1	-0.05 (-0.18 to 0.08)	-0.21 (-0.37 to -0.05)	-0.33 (-0.51 to -0.15)	-0.36 (-0.53 to -0.19)
Protein S free	23.6	0.00 (-0.12 to 0.12)	-0.29 (-0.43 to -0.15)	0.05 (-0.11 to 0.21)	-0.03 (-0.18 to 0.12)
Antithrombin III	10.7	-0.43 (-0.52 to -0.34)	-0.27 (-0.38 to -0.16)	-0.11 (-0.23 to 0.02)	-0.19 (-0.30 to -0.07)
Factor II activity	12.3	0.53 (0.39 to 0.68)	0.22 (0.05 to 0.40)	0.25 (0.06 to 0.45)	0.24 (0.06 to 0.42)
Factor VIII activity	38.2	0.08 (-0.01 to 0.17)	0.17 (0.07 to 0.28)	0.15 (0.03 to 0.27)	0.24 (0.13 to 0.36)
Factor IX activity	39.3	0.01 (-0.08 to 0.10)	-0.06 (-0.17 to 0.05)	0.03 (-0.09 to 0.15)	-0.14 (-0.26 to -0.03)
Factor XI activity	30.2	0.11 (0.03 to 0.20)	0.10 (0.00 to 0.21)	0.09 (-0.03 to 0.21)	0.10 (-0.02 to 0.21)
Fibrinogen activity	0.70	0.16 (0.06 to 0.26)	-0.03 (-0.15 to 0.10)	0.12 (-0.01 to 0.26)	0.19 (0.06 to 0.32)
Age at date of index	11.8	-0.10 (-0.19 to 0.00)	-0.12 (-0.24 to 0.00)	-0.03 (-0.16 to 0.10)	-0.02 (-0.14 to 0.10)
Sex*		-0.09 (-0.27 to 0.08)	0.18 (-0.03 to 0.39)	0.13 (-0.11 to 0.37)	0.10 (-0.12 to 0.33)
Use of contraceptives*		0.12 (-0.01 to 0.26)	0.07 (-0.10 to 0.24)	-0.07 (-0.25 to 0.12)	0.02 (-0.16 to 0.19)
Smoking habits*		0.04 (-0.02 to 0.10)	-0.05 (-0.13 to 0.02)	0.07 (-0.02 to 0.15)	0.08 (0.01 to 0.16)

*Dichotomous values, not standardized.

Addition of CTI changed the determinants to some extent. The association for some coagulation proteins became stronger when measuring TG in plasma with CTI, i.e., PS for the CAT assay (Z-score changed from 0.00 (-0.12 to 0.12) to -0.29 (-0.43 to -0.15) for free PS and from -0.05 (-0.18 to 0.08) to -0.21 (-0.37 to -0.05) for PS activity) and AT, FVIII and Fibrinogen for the TGA (Z-score changed from -0.11 (-0.23 to 0.02) to -0.19 (-0.30 to -0.07) for AT, from 0.15 (0.03 to 0.27) to 0.24 (0.13 to 0.36) for FVIII, and from 0.12 (-0.01 to 0.26) to 0.19 (0.06 to 0.32) for fibrinogen. In contrast with the standard samples, some determinants did not seem to be associated with TG in the presence of CTI (PC in both assays and fibrinogen in the CAT assay). In the TGA, FIX became associated with TG only after the addition of CTI, but not in the direction as expected (standardized β: -0.14; -0.26 to -0.03).

The results of multivariate analysis of standardized values of the remaining TG parameters, such as peak height, are shown in S1 and S2 Tables. The parameters, especially peak height, show a similar results as ETP/AUC.

## Discussion

In our study we aimed to establish the effect of CTI on the contribution of individual coagulation factors to thrombin generation measured with the CAT and TGA assay. As expected, thrombin generation decreased after addition of CTI, i.e., in both assays peak height and ETP/AUC decreased clearly by CTI addition. However, there was not a trend towards a broader contribution of coagulation proteins to TG in the samples with CTI compared with samples without CTI.

In accordance with previous studies, we observed that addition of CTI to both CAT and TGA assays decreased the peak height and ETP/AUC [5, 7–10, 15]. This indicates that thrombin generated due to contact activation, i.e. in-vitro artefacts, are removed by CTI. CTI inhibits contact activation completely by irreversible binding and inactivation of FXIIa [2, 7, 8, 15, 16]. I.e., for precise measurement of TG, addition of CTI is therefore useful also as it allows initiation using a low TF trigger concentration. However, we did not find that the addition of CTI was associated with more and stronger determinants of TG assay. This is in accordance with our previous study, where CTI did not alter the association between thrombin generation measurement and a first VTE or recurrent VTE [10].

We showed that addition of CTI changes the determinants of TG minimally. While the association for some coagulation proteins became stronger when measuring TG in plasma with CTI (i.e., PS for the CAT assay and AT, FVIII and fibrinogen for the TGA) some determinants did not seem to be associated with TG in the presence of CTI (PC in both assays and fibrinogen in the CAT assay). In literature, ATIII, protein C, FVIII and fibrinogen are known determinants of TG in general [1, 17, 18]. This is in accordance with our findings in samples without CTI. However, we found that fibrinogen and PC were no longer determinants after addition of CTI. For protein C this was striking, as it was observed in both assays (for fibrinogen in the CAT only). In part, this may be explained by the fact that we did not add thrombomodulin (TM) to the measurements, which is a cofactor in the protein C system [19]. However, the finding that PC was a determinant in absence of TM, in the samples without CTI, is in contrast with previous publications. Specifically for CAT assay, besides PS, also free TFPI has been identified as a determinant of TG [20]. Unfortunately, TFPI was not measured in this study population.

To our knowledge, only Dielis and colleagues identified determinants of TG with addition of CTI. They measured TG in the normal population using the CAT at the same TF concentration (1pM). However, they did not repeat their measurements without CTI addition in order to compare the results [21]. They also found that ATIII and free protein S are major determinants of TG, however, free protein S only contributed to lag time in their study. Furthermore, they showed that with CTI, protein C contributed to TG when thrombomodulin was added and disappeared when thrombomodulin was not added. The latter result was in accordance with our findings. Finally, they identified FXII as a major determinant despite removal of contact activation [21]. We did not measure FXII, however, the contribution of FXI to thrombin generation only marginally changed after inhibition of FXIIa by CTI. This finding may be explained by the fact that FXI is also activated by thrombin in the context of amplification of thrombin, i.e. propagation phase of thrombin generation in which FXI plays an essential role. This contribution appears to be most important in case of low TF concentrations [22].

A strength of our study is the relatively large sample size. Furthermore, we measured a wide range of coagulation determinants, including both pro- and anticoagulant factors. We used a very low TF trigger concentration, which enabled us to study the role of many coagulation factors in thrombin generation coagulation in vivo. The choice to study healthy individuals only is a strength of our study, as this avoids spurious associations between thrombin generation and individual coagulation factors due to index event bias [23]. Our study also has some limitations. For the comparison between standard and optimized samples, different TF trigger concentrations were used (0.5pM, 1pM and 5pM). Although all concentrations are very low, they could theoretically have hampered our comparisons. Furthermore, the sample size was relatively small but sufficient for the performed analyses.

An important limitation of our study was that TFPI was not measured in the study population, although in literature it was described as an important determinant of the CAT assay. It would have been informative to know whether this remains after addition of CTI. However, the current study focusses on the research question whether TG becomes a more global test after addition of CTI. This would be the case if more and stronger determinants of TG assay would be found in the presence of CTI. Since TFPI was already an important determinant of CAT conducted in the absence of CTI, addition of TFPI to our results would not change our conclusion. TFPI would, at most, remain an important determinant of the CAT assay in the presence of CTI, which is very likely. Regarding the TGA assay, it is unknown whether TFPI is an important determinant of TG without CTI, nor do we know if this remains in presence of CTI.

Another limitation is the effect of alpha-2-macroglobulin in TGA. In contrast to TGA, in CAT a software correction for the effect of alpha-2-macroglobulin on thrombin generation was applied. However, as the effect of alpha-2-macroglobulin on thrombin generation has previously been shown to be minimal [24], we expect the influence on the validity of our findings to be minimal as well. For the optimized samples, blood was sampled in CTI-coated vacutainers, as this is shown to neutralize FXIIa completely [5], instead of adding CTI at a later stage after blood sampling. Sampling was performed using minimal suction and a light tourniquet. Despite the notion that free-flowing blood without a tourniquet would be optimal, it was practically not feasible. Laboratory staff were experienced in thrombin generation testing and the protocol was firmly adhered to.

In conclusion, CTI improves the precision of TG measurements for both CAT and TGA assays. However, when comparing the contribution of individual coagulation factors to thrombin generation using low TF trigger concentration, there is not a trend towards a broader contribution of coagulation proteins in the samples with CTI compared with samples without CTI.

## Supporting information

S1 Table Mean change in thrombin generation parameter with 1 SD increase in coagulation factor for CAT. (DOCX)

S2 Table Mean change in thrombin generation parameter with 1 SD increase in coagulation factor for TGA. (DOCX)
