# Supplementary material for: The influence of corn trypsin inhibitor on the contribution of coagulation determinants to the Technoclone Thrombin Generation Assay (TGA) and the Calibrated Automated Thrombogram (CAT)
Source: PLoS One. 2022 Feb 25;17(2):e0263960. doi: 10.1371/journal.pone.0263960 (PMC8880747; doi:10.1371/journal.pone.0263960)
Supplement: S1 Table — (DOCX) [file pone.0263960.s001.docx]

**S1 Table.** **Mean change in thrombin generation parameter with 1 SD increase in coagulation factor for CAT**. Including sex (1 = male, 2 = female), age, use of contraceptives (0 = no, 1 = yes), and smoking habits (0 = smoked never, or ceased smoking before inclusion, 1 = smoking).

|  | SD | CAT standardized B (95%CI) | | | | | | | |
| --- | --- | --- | --- | --- | --- | --- | --- | --- | --- |
|  |  | Lag time (Citrate)  (SD = 0.44) | Lag time (CTI)  (SD = 1.46) | Time to peak (Citrate)  (SD = 175.54) | Time to peak (CTI)  (SD = 2.26) | Peak (Citrate)  (SD = 61.75) | Peak (CTI)  (SD = 39.79) | ETP (Citrate)  (SD 318.78) | ETP (CTI)  (SD = 341.58) |
| (Z-score) Protein C activity (%) | 23.6 | -0.01 (-0.13 to 0.11) | 0.05 (-0.06 to 0.16) | -0.03 (-0.19 to 0.14) | 0.02 (-0.10 to 0.14) | 0.09 (-0.03 to 0.21) | -0.01 (-0.14 to 0.12) | 0.10 (0.00 to 0.21) | -0.06 (-0.19 to 0.06) |
| (Z-score) Protein S activity (%) | 15.1 | -0.04 (-0.20 to 0.11) | -0.07 (-0.21 to 0.07) | 0.06 (-0.15 to 0.28) | -0.10 (-0.25 to 0.05) | -0.07 (-0.22 to 0.08) | -0.27 (-0.43 to -0.10) | -0.05 (-0.18 to 0.08) | -0.21 (-0.37 to -0.05) |
| (Z-score) Protein S free (%) | 23.6 | 0.19 (0.05 to 0.32) | 0.24 (0.11 to 0.36) | 0.01 (-0.17 to 0.20) | 0.28 (0.15 to 0.41) | -0.20 (-0.33 to -0.06) | -0.29 (-0.43 to -0.14) | 0.00 (-0.12 to 0.12) | -0.29 (-0.43 to -0.15) |
| (Z-score) Antithrombin III (%) | 10.7 | 0.01 (-0.10 to 0.12) | 0.06 (-0.04 to 0.16) | 0.03 (-0.11 to 0.18) | 0.05 (-0.05 to 0.16) | -0.18 (-0.28 to -0.07) | -0.21 (-0.33 to -0.10) | -0.43 (-0.52 to -0.34) | -0.27 (-0.38 to -0.16) |
| (Z-score) Factor II activity (%) | 12.3 | 0.02 (-0.15 to 0.18) | 0.06 (-0.09 to 0.21) | -0.18 (-0.41 to 0.05) | 0.17 (0.01 to 0.33) | 0.34 (0.18 to 0.51) | 0.11 (-0.07 to 0.28) | 0.53 (0.39 to 0.68) | 0.22 (0.05 to 0.40) |
| (Z-score) Factor VIII activity (%) | 38.2 | 0.02 (-0.09 to 0.12) | 0.11 (0.01 to 0.20) | 0.11 (-0.03 to 0.25) | -0.05 (-0.15 to 0.05) | 0.15 (0.05 to 0.25) | 0.29 (0.18 to 0.40) | 0.08 (-0.01 to 0.17) | 0.17 (0.07 to 0.28) |
| (Z-score) Factor IX activity (%) | 39.3 | -0.09 (-0.19 to 0.02) | 0.06 (-0.03 to 0.15) | -0.04 (-0.18 to 0.11) | 0.04 (-0.06 to 0.14) | 0.09 (-0.01 to 0.19) | 0.01 (-0.10 to 0.12) | 0.01 (-0.08 to 0.10) | -0.06 (-0.17 to 0.05) |
| (Z-score) Factor XI activity (%) | 30.2 | 0.01 (-0.10 to 0.11) | -0.01 (-0.10 to 0.08) | 0.03 (-0.11 to 0.17) | 0.04 (-0.06 to 0.14) | 0.07 (-0.03 to 0.17) | 0.07 (-0.04 to 0.19) | 0.11 (0.03 to 0.20) | 0.10 (0.00 to 0.21) |
| (Z-score) Fibrinogen (mg/dL) | 0.70 | 0.26 (0.15 to 0.38) | 0.45 (0.34 to 0.55) | 0.00 (-0.16 to 0.16) | 0.41 (0.29 to 0.52) | 0.04 (-0.07 to 0.16) | -0.09 (-0.21 to 0.04) | 0.16 (0.06 to 0.26) | -0.03 (-0.15 to 0.10) |
| Sex | NA | -0.11 (-0.32 to 0.09) | -0.27 (-0.45 to -0.08) | 0.10 (-0.19 to 0.38) | -0.28 (-0.48 to -0.08) | 0.02 (-0.18 to 0.22) | 0.16 (-0.06 to 0.38) | -0.09 (-0.27 to 0.08) | 0.18 (-0.03 to 0.39) |
| (Z-score) Age at date of index (years) | 11.8 | 0.03 (-0.08 to 0.14) | 0.03 (-0.07 to 0.13) | 0.00 (-0.16 to 0.15) | 0.10 (-0.01 to 0.21) | -0.06 (-0.17 to 0.05) | -0.14 (-0.26 to -0.02) | -0.10 (-0.19 to 0.00) | -0.12 (-0.24 to 0.00) |
| Use of contraceptives | NA | 0.09 (-0.07 to 0.25) | 0.01 (-0.14 to 0.15) | 0.02 (-0.20 to 0.23) | 0.05 (-0.10 to 0.20) | 0.15 (0.00 to 0.31) | 0.12 (-0.06 to 0.29) | 0.12 (-0.01 to 0.26) | 0.07 (-0.10 to 0.24) |
| Smoking habits | NA | 0.01 (-0.06 to 0.09) | -0.02 (-0.09 to 0.04) | -0.01 (-0.11 to 0.09) | 0.03 (-0.04 to 0.10) | 0.02 (-0.05 to 0.09) | -0.08 (-0.15 to 0.00) | 0.04 (-0.02 to 0.10) | -0.05 (-0.13 to 0.02) |

*Abbreviations*: CI, confidence interval; SD, standard deviation; ETP, endogenous thrombin potential; CAT, calibrated automated thrombogram; CTI, corn trypsin inhibitor.
